# (*E*)-3-(4-Methyl­phen­yl)-1-(4-nitro­phenyl)prop-2-en-1-one

**DOI:** 10.1107/S1600536808012257

**Published:** 2008-05-03

**Authors:** Hoong-Kun Fun, Suchada Chantrapromma, P. S. Patil, E. Deepak D’Silva, S. M. Dharmaprakash

**Affiliations:** a X-ray Crystallography Unit, School of Physics, Universiti Sains Malaysia, 11800 USM, Penang, Malaysia; b Department of Chemistry, Faculty of Science, Prince of Songkla University, Hat-Yai, Songkhla 90112, Thailand; c Department of Studies in Physics, Mangalore University, Mangalagangotri, Mangalore 574 199, India

## Abstract

The asymmetric unit of the title compound, C_16_H_13_NO_3_, contains two independent mol­ecules related approximately by a pseudo-twofold rotation axis. The dihedral angle between the nitro­benzene and methyl­phenyl rings is 42.18 (6)° in one mol­ecule and 12.97 (6)° in the other. In both mol­ecules, the nitro group is slightly twisted away from the attached benzene ring. In the crystal structure, the mol­ecules are stacked along the *b* axis and are linked *via* C—H⋯O and C—H⋯π inter­actions.

## Related literature

For bond-length data, see: Allen *et al.* (1987 ▶). For hydrogen-bond motifs, see: Bernstein *et al.* (1995 ▶). For related structures, see: Fun *et al.* (2007 ▶); Patil *et al.* (2007 ▶ **a* ▶,b* ▶); Patil, Dharmaprakash *et al.* (2007 ▶). For background to the applications of substituted chalcones, see: Agrinskaya *et al.* (1999 ▶); Gu *et al.* (2008 ▶); Patil *et al.* (2006 ▶); Patil, Dharmaprakash *et al.* (2007 ▶).
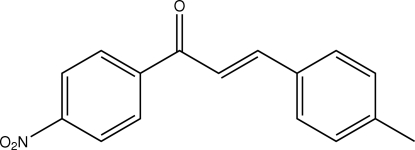


## Experimental

### 

#### Crystal data

C_16_H_13_NO_3_
*M* *_r_* = 267.27
Triclinic, 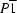

*a* = 5.8857 (1) Å
*b* = 7.8800 (1) Å
*c* = 27.4745 (4) Å
α = 88.793 (1)°
β = 85.665 (1)°
γ = 82.645 (1)°
*V* = 1260.07 (3) Å^3^
*Z* = 4
Mo *K*α radiation
μ = 0.10 mm^−1^
*T* = 100.0 (1) K
0.43 × 0.26 × 0.23 mm

#### Data collection

Bruker SMART APEXII CCD area-detector diffractometer
Absorption correction: multi-scan (*SADABS*; Bruker, 2005 ▶) *T*_min_ = 0.959, *T*_max_ = 0.977
32268 measured reflections
6666 independent reflections
5182 reflections with *I* > 2σ(*I*)
*R*_int_ = 0.033

#### Refinement

*R*[*F* ^2^ > 2σ(*F* ^2^)] = 0.047
*wR*(*F* ^2^) = 0.131
*S* = 1.09
6666 reflections
363 parameters
H-atom parameters constrained
Δρ_max_ = 0.38 e Å^−3^
Δρ_min_ = −0.26 e Å^−3^

### 

Data collection: *APEX2* (Bruker, 2005 ▶); cell refinement: *APEX2*; data reduction: *SAINT* (Bruker, 2005 ▶); program(s) used to solve structure: *SHELXTL* (Sheldrick, 2008 ▶); program(s) used to refine structure: *SHELXTL*; molecular graphics: *SHELXTL*; software used to prepare material for publication: *SHELXTL* and *PLATON* (Spek, 2003 ▶).

## Supplementary Material

Crystal structure: contains datablocks global, I. DOI: 10.1107/S1600536808012257/ci2586sup1.cif

Structure factors: contains datablocks I. DOI: 10.1107/S1600536808012257/ci2586Isup2.hkl

Additional supplementary materials:  crystallographic information; 3D view; checkCIF report

## Figures and Tables

**Table 1:** Hydrogen-bond geometry (Å, °)

*D*—H⋯*A*	*D*—H	H⋯*A*	*D*⋯*A*	*D*—H⋯*A*
C1*A*—H1*A*⋯O1*B*^i^	0.93	2.58	3.2597 (17)	131
C9*A*—H9*A*⋯O1*A*	0.93	2.48	2.8045 (17)	101
C9*B*—H9*B*⋯O1*B*	0.93	2.48	2.8112 (17)	101
C1*B*—H1*B*⋯*Cg*1	0.93	2.90	3.4853 (15)	123
C4*B*—H4*B*⋯*Cg*1^ii^	0.93	2.86	3.4837 (15)	126
C16*A*—H16*C*⋯*Cg*2^iii^	0.96	2.91	3.7837 (15)	151

Symmetry codes: (i) 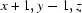; (ii) 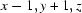; (iii) 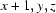. *Cg*1 and *Cg*2 are centroids of the C10*A*–C15*A* and C1*B*–C6*B* rings, ,respectively.

**Fig. 1.**
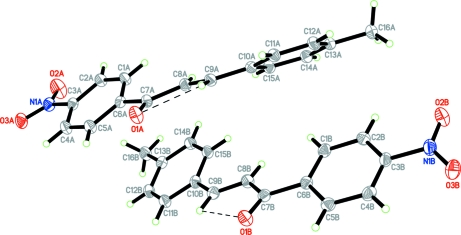
The asymmetric unit of the title compound, showing 50% probability displacement ellipsoids and the atomic numbering. Weak C—H···O intramolecular interactions are drawn as dashed lines.

**Fig. 2.**
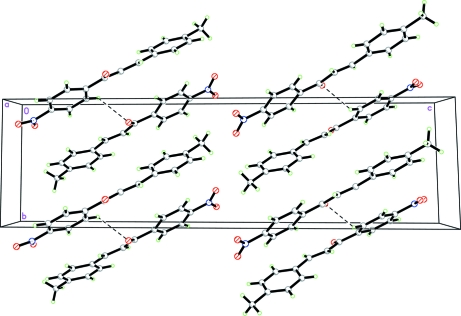
The crystal packing of the title compound, viewed along the a axis showing stacking of anti-parallel pairs of molecules approximately along the b axis. Hydrogen bonds are drawn as dashed lines.

## supplementary crystallographic information

### Comment

Substituted chalcones exhibit second-harmonic generation in crystalline form and possess optical limiting behavior with femtosecond laser pulse at 780 nm wavelength (Gu et al., 2008; Patil et al., 2006, 2007c; Agrinskaya et al., 1999). The main idea behind the above studies was to introduce various donor/acceptor substituents [OCH_3_, N(CH_3_)_2_, NH_2_, F, Cl, Br, CH_3_, NO_2_] on either side of benzene rings and to observe the structure-activity relationship. In view of the importance  of substituted chalcones, the title compound was synthesized and its crystal structure is reported here.

There are two independent molecules, A and B, in the asymmetric unit of the title compound (Fig. 1). Bond lengths and angles in both molecules are in normal ranges (Allen et al., 1987) and comparable to those in related structures (Fun et al., 2007; Patil et al., 2007a,b). The dihedral angles between the nitrobenzene and methylphenyl rings are 42.18 (6)° and 12.97 (6)° in molecule A and B, respectively. In molecule A, atoms O1A, C6A, C7A and C8A are coplanar and the least-squares plane through these atoms makes dihedral angles of 20.21 (8)° and 24.41 (7)° with the nitrobenzene (C1A–C6A) and methylbenzene (C10A–C15A) rings, repectively. However, in molecule B atoms O1B, C6B, C7B, C8B, C9B and C10B are coplanar, and the dihedral angles formed by the mean plane through these atoms with the nirobenzene and methylbenzene rings are 16.85 (6)° and 16.97 (6)°, respectively. The nitro groups are slightly twisted away from the plane of the attached benzene rings, with the O2—N1—C3—C2 torsion angles being 11.2 (2)° and 5.84 (19)° in molecules A and B, respectively, and the O3—N1—C3—C4 torsion angles being 11.5 (2)° and 4.54 (19)°, in A and B,respectively. In each of the independent molecules, a weak C9—H9···O1 interaction generates an S(5) ring motif (Bernstein et al.,  1995) (Table 1).

In the crystal structure (Fig. 2), the molecules are stacked in as anti-parallel pairs approximately along the b axis. The crystal structure is stabilized by weak C—H···O hydrogen bonds and C—H···π interactions (Table 1) involving the C10A-C15A (centroid Cg1) and C1B-C6B (centroid Cg2) benzene rings.

### Experimental

The title compound was synthesized by the condensation of *p*-tolualdehyde (0.01 mol) with 4-nitroacetophenone (0.01 mol) in methanol (60 ml) in the presence of a catalytic amount of sodium hydroxide solution (5 ml, 30%). After stirring for 2 hr, the contents of the flask were poured into ice-cold water (500 ml) and left to stand for 5 hr. The resulting crude solid was filtered and dried. Yellow single crystals of the title compound suitable for X-ray structure determination were recrystallized from *N*,*N*-dimethylformamide (DMF).

### Refinement

All H atoms were placed in calculated positions, with d(C—H) = 0.93 Å, *U*_iso_(H) = 1.2*U*_eq_(C) for and aromatic H and d(C—H) = 0.96 Å, *U*_iso_(H) = 1.5*U*_eq_(C) for methyl H atoms. A rotating group model was used for the methyl groups.

### Crystal data

**Table:**

C_16_H_13_NO_3_	*Z* = 4
*M**_r_* = 267.27	*F*_000_ = 560
Triclinic, *P*1	*D*_x_ = 1.409 Mg m^−^^3^
Hall symbol: -P 1	Mo *K*α radiation λ = 0.71073 Å
*a* = 5.8857 (1) Å	Cell parameters from 6666 reflections
*b* = 7.8800 (1) Å	θ = 0.7–29.0º
*c* = 27.4745 (4) Å	µ = 0.10 mm^−^^1^
α = 88.793 (1)º	*T* = 100.0 (1) K
β = 85.665 (1)º	Block, yellow
γ = 82.645 (1)º	0.43 × 0.26 × 0.23 mm
*V* = 1260.07 (3) Å^3^	

### Data collection

**Table:**

Bruker SMART APEXII CCD area-detector diffractometer	6666 independent reflections
Radiation source: fine-focus sealed tube	5182 reflections with *I* > 2σ(*I*)
Monochromator: graphite	*R*_int_ = 0.033
Detector resolution: 8.33 pixels mm^-1^	θ_max_ = 29.0º
*T* = 100.0(1) K	θ_min_ = 0.7º
ω scans	*h* = −8→8
Absorption correction: multi-scan(SADABS; Bruker, 2005)	*k* = −10→8
*T*_min_ = 0.959, *T*_max_ = 0.977	*l* = −37→37
32268 measured reflections	

### Refinement

**Table:**

Refinement on *F*^2^	Secondary atom site location: difference Fourier map
Least-squares matrix: full	Hydrogen site location: inferred from neighbouring sites
*R*[*F*^2^ > 2σ(*F*^2^)] = 0.047	H-atom parameters constrained
*wR*(*F*^2^) = 0.131	*w* = 1/[σ^2^(*F*_o_^2^) + (0.061*P*)^2^ + 0.3735*P*] where *P* = (*F*_o_^2^ + 2*F*_c_^2^)/3
*S* = 1.09	(Δ/σ)_max_ = 0.001
6666 reflections	Δρ_max_ = 0.38 e Å^−^^3^
363 parameters	Δρ_min_ = −0.26 e Å^−^^3^
Primary atom site location: structure-invariant direct methods	Extinction correction: none

### Special details

**Table:**

Experimental. The low-temperature data was collected with the Oxford Cyrosystem Cobra low-temperature attachment.
Geometry. All e.s.d.'s (except the e.s.d. in the dihedral angle between two l.s. planes) are estimated using the full covariance matrix. The cell e.s.d.'s are taken into account individually in the estimation of e.s.d.'s in distances, angles and torsion angles; correlations between e.s.d.'s in cell parameters are only used when they are defined by crystal symmetry. An approximate (isotropic) treatment of cell e.s.d.'s is used for estimating e.s.d.'s involving l.s. planes.
Refinement. Refinement of *F*^2^ against ALL reflections. The weighted *R*-factor *wR* and goodness of fit *S* are based on *F*^2^, conventional *R*-factors *R* are based on *F*, with *F* set to zero for negative *F*^2^. The threshold expression of *F*^2^ > σ(*F*^2^) is used only for calculating *R*-factors(gt) *etc*. and is not relevant to the choice of reflections for refinement. *R*-factors based on *F*^2^ are statistically about twice as large as those based on *F*, and *R*- factors based on ALL data will be even larger.

### Fractional atomic coordinates and isotropic or equivalent isotropic displacement parameters (Å^2^)

**Table:**

	*x*	*y*	*z*	*U*_iso_*/*U*_eq_	
O1A	0.21638 (17)	0.20887 (14)	0.77198 (4)	0.0239 (2)	
O2A	0.88278 (19)	−0.18446 (17)	0.96280 (4)	0.0382 (3)	
O3A	0.52447 (19)	−0.18264 (14)	0.98705 (4)	0.0271 (2)	
N1A	0.6782 (2)	−0.14787 (16)	0.95730 (4)	0.0219 (3)	
C1A	0.7223 (2)	0.03671 (18)	0.83164 (5)	0.0193 (3)	
H1A	0.8340	0.0463	0.8063	0.023*	
C2A	0.7813 (2)	−0.04744 (18)	0.87474 (5)	0.0196 (3)	
H2A	0.9319	−0.0955	0.8785	0.024*	
C3A	0.6122 (2)	−0.05826 (17)	0.91193 (5)	0.0171 (3)	
C4A	0.3867 (2)	0.00997 (18)	0.90810 (5)	0.0203 (3)	
H4A	0.2761	0.0009	0.9337	0.024*	
C5A	0.3298 (2)	0.09255 (18)	0.86483 (5)	0.0197 (3)	
H5A	0.1786	0.1394	0.8613	0.024*	
C6A	0.4958 (2)	0.10657 (17)	0.82647 (5)	0.0158 (3)	
C7A	0.4201 (2)	0.19297 (17)	0.77999 (5)	0.0169 (3)	
C8A	0.5949 (2)	0.25902 (17)	0.74603 (5)	0.0174 (3)	
H8A	0.7450	0.2555	0.7548	0.021*	
C9A	0.5374 (2)	0.32427 (17)	0.70247 (5)	0.0170 (3)	
H9A	0.3891	0.3156	0.6941	0.020*	
C10A	0.6854 (2)	0.40782 (17)	0.66680 (5)	0.0158 (3)	
C11A	0.6179 (2)	0.43784 (17)	0.61919 (5)	0.0172 (3)	
H11A	0.4811	0.4026	0.6109	0.021*	
C12A	0.7501 (2)	0.51888 (17)	0.58421 (5)	0.0185 (3)	
H12A	0.7027	0.5350	0.5527	0.022*	
C13A	0.9536 (2)	0.57638 (17)	0.59585 (5)	0.0179 (3)	
C14A	1.0189 (2)	0.54932 (17)	0.64355 (5)	0.0181 (3)	
H14A	1.1525	0.5887	0.6521	0.022*	
C15A	0.8900 (2)	0.46549 (17)	0.67837 (5)	0.0177 (3)	
H15A	0.9396	0.4474	0.7096	0.021*	
C16A	1.0971 (2)	0.66602 (19)	0.55834 (5)	0.0224 (3)	
H16A	0.9997	0.7509	0.5414	0.034*	
H16B	1.1753	0.5841	0.5354	0.034*	
H16C	1.2079	0.7203	0.5743	0.034*	
O1B	−0.02096 (17)	0.84372 (13)	0.73497 (4)	0.0228 (2)	
O2B	0.65858 (19)	1.10645 (15)	0.52474 (4)	0.0302 (3)	
O3B	0.3249 (2)	1.25845 (15)	0.52514 (4)	0.0342 (3)	
N1B	0.4637 (2)	1.14949 (16)	0.54285 (4)	0.0227 (3)	
C1B	0.4905 (2)	0.88516 (18)	0.65652 (5)	0.0193 (3)	
H1B	0.5979	0.8123	0.6726	0.023*	
C2B	0.5542 (2)	0.95813 (18)	0.61183 (5)	0.0196 (3)	
H2B	0.7030	0.9339	0.5976	0.024*	
C3B	0.3911 (2)	1.06743 (17)	0.58920 (5)	0.0181 (3)	
C4B	0.1681 (2)	1.10666 (18)	0.60879 (5)	0.0204 (3)	
H4B	0.0625	1.1817	0.5928	0.024*	
C5B	0.1060 (2)	1.03113 (18)	0.65291 (5)	0.0192 (3)	
H5B	−0.0441	1.0541	0.6665	0.023*	
C6B	0.2662 (2)	0.92085 (17)	0.67722 (5)	0.0167 (3)	
C7B	0.1851 (2)	0.84063 (17)	0.72448 (5)	0.0175 (3)	
C8B	0.3587 (2)	0.75994 (18)	0.75670 (5)	0.0190 (3)	
H8B	0.5139	0.7541	0.7466	0.023*	
C9B	0.2950 (2)	0.69516 (17)	0.80024 (5)	0.0186 (3)	
H9B	0.1380	0.7050	0.8089	0.022*	
C10B	0.4455 (2)	0.61059 (17)	0.83569 (5)	0.0176 (3)	
C11B	0.3559 (2)	0.57945 (18)	0.88324 (5)	0.0196 (3)	
H11B	0.2014	0.6141	0.8919	0.024*	
C12B	0.4946 (2)	0.49752 (18)	0.91766 (5)	0.0203 (3)	
H12B	0.4320	0.4792	0.9491	0.024*	
C13B	0.7260 (2)	0.44249 (18)	0.90559 (5)	0.0194 (3)	
C14B	0.8147 (2)	0.47184 (18)	0.85790 (5)	0.0197 (3)	
H14B	0.9684	0.4348	0.8490	0.024*	
C15B	0.6776 (2)	0.55527 (18)	0.82360 (5)	0.0192 (3)	
H15B	0.7408	0.5746	0.7922	0.023*	
C16B	0.8747 (3)	0.3557 (2)	0.94323 (5)	0.0236 (3)	
H16D	0.8475	0.4184	0.9731	0.035*	
H16E	1.0334	0.3520	0.9317	0.035*	
H16F	0.8385	0.2413	0.9489	0.035*	

### Atomic displacement parameters (Å^2^)

**Table:**

	*U*^11^	*U*^22^	*U*^33^	*U*^12^	*U*^13^	*U*^23^
O1A	0.0172 (5)	0.0334 (6)	0.0213 (5)	−0.0041 (4)	−0.0029 (4)	0.0080 (4)
O2A	0.0253 (6)	0.0570 (8)	0.0311 (6)	0.0002 (5)	−0.0093 (5)	0.0183 (6)
O3A	0.0333 (6)	0.0294 (6)	0.0187 (5)	−0.0066 (5)	0.0006 (4)	0.0073 (4)
N1A	0.0267 (6)	0.0219 (6)	0.0173 (6)	−0.0033 (5)	−0.0037 (5)	0.0040 (5)
C1A	0.0173 (6)	0.0225 (7)	0.0174 (6)	−0.0017 (5)	0.0016 (5)	0.0022 (5)
C2A	0.0172 (6)	0.0226 (7)	0.0187 (7)	−0.0005 (5)	−0.0023 (5)	0.0035 (5)
C3A	0.0205 (7)	0.0170 (6)	0.0143 (6)	−0.0036 (5)	−0.0030 (5)	0.0023 (5)
C4A	0.0190 (7)	0.0243 (7)	0.0175 (6)	−0.0040 (5)	0.0014 (5)	0.0032 (5)
C5A	0.0165 (6)	0.0232 (7)	0.0187 (6)	−0.0009 (5)	−0.0009 (5)	0.0037 (5)
C6A	0.0171 (6)	0.0146 (6)	0.0161 (6)	−0.0034 (5)	−0.0014 (5)	0.0010 (5)
C7A	0.0186 (6)	0.0172 (6)	0.0147 (6)	−0.0024 (5)	−0.0002 (5)	0.0017 (5)
C8A	0.0159 (6)	0.0177 (6)	0.0184 (6)	−0.0025 (5)	0.0000 (5)	0.0012 (5)
C9A	0.0159 (6)	0.0181 (6)	0.0166 (6)	−0.0015 (5)	−0.0002 (5)	0.0005 (5)
C10A	0.0166 (6)	0.0146 (6)	0.0155 (6)	−0.0002 (5)	0.0001 (5)	0.0012 (5)
C11A	0.0163 (6)	0.0178 (6)	0.0176 (6)	−0.0027 (5)	−0.0010 (5)	−0.0002 (5)
C12A	0.0223 (7)	0.0190 (7)	0.0132 (6)	−0.0001 (5)	−0.0004 (5)	0.0014 (5)
C13A	0.0190 (6)	0.0149 (6)	0.0184 (6)	0.0005 (5)	0.0028 (5)	0.0013 (5)
C14A	0.0150 (6)	0.0189 (7)	0.0204 (7)	−0.0028 (5)	−0.0001 (5)	−0.0006 (5)
C15A	0.0185 (6)	0.0187 (7)	0.0155 (6)	−0.0010 (5)	−0.0011 (5)	0.0005 (5)
C16A	0.0235 (7)	0.0222 (7)	0.0208 (7)	−0.0040 (6)	0.0038 (5)	0.0027 (5)
O1B	0.0193 (5)	0.0286 (6)	0.0201 (5)	−0.0027 (4)	−0.0007 (4)	0.0026 (4)
O2B	0.0307 (6)	0.0363 (6)	0.0235 (5)	−0.0083 (5)	0.0044 (4)	0.0055 (5)
O3B	0.0455 (7)	0.0300 (6)	0.0249 (6)	0.0027 (5)	−0.0036 (5)	0.0107 (5)
N1B	0.0318 (7)	0.0202 (6)	0.0169 (6)	−0.0065 (5)	−0.0013 (5)	0.0020 (5)
C1B	0.0185 (6)	0.0180 (7)	0.0209 (7)	−0.0001 (5)	−0.0033 (5)	0.0033 (5)
C2B	0.0173 (6)	0.0197 (7)	0.0212 (7)	−0.0014 (5)	0.0009 (5)	−0.0002 (5)
C3B	0.0255 (7)	0.0152 (6)	0.0140 (6)	−0.0045 (5)	−0.0020 (5)	0.0012 (5)
C4B	0.0227 (7)	0.0179 (7)	0.0201 (7)	0.0006 (5)	−0.0052 (5)	0.0019 (5)
C5B	0.0181 (6)	0.0191 (7)	0.0199 (7)	−0.0002 (5)	−0.0016 (5)	0.0002 (5)
C6B	0.0187 (6)	0.0160 (6)	0.0156 (6)	−0.0029 (5)	−0.0022 (5)	0.0001 (5)
C7B	0.0191 (6)	0.0163 (6)	0.0170 (6)	−0.0021 (5)	−0.0015 (5)	−0.0004 (5)
C8B	0.0176 (6)	0.0206 (7)	0.0187 (7)	−0.0014 (5)	−0.0022 (5)	0.0017 (5)
C9B	0.0193 (6)	0.0181 (7)	0.0184 (6)	−0.0020 (5)	−0.0016 (5)	0.0008 (5)
C10B	0.0204 (7)	0.0177 (6)	0.0155 (6)	−0.0047 (5)	−0.0028 (5)	0.0018 (5)
C11B	0.0181 (6)	0.0225 (7)	0.0179 (6)	−0.0024 (5)	−0.0003 (5)	0.0024 (5)
C12B	0.0234 (7)	0.0222 (7)	0.0152 (6)	−0.0036 (6)	−0.0010 (5)	0.0035 (5)
C13B	0.0215 (7)	0.0175 (7)	0.0198 (7)	−0.0043 (5)	−0.0032 (5)	0.0011 (5)
C14B	0.0179 (6)	0.0214 (7)	0.0194 (7)	−0.0015 (5)	−0.0012 (5)	0.0018 (5)
C15B	0.0226 (7)	0.0200 (7)	0.0150 (6)	−0.0040 (5)	−0.0001 (5)	0.0012 (5)
C16B	0.0237 (7)	0.0290 (8)	0.0177 (7)	−0.0020 (6)	−0.0027 (5)	0.0057 (6)

### Geometric parameters (Å, °)

**Table:**

O1A—C7A	1.2256 (16)	O1B—C7B	1.2224 (17)
O2A—N1A	1.2219 (16)	O2B—N1B	1.2245 (16)
O3A—N1A	1.2263 (16)	O3B—N1B	1.2264 (16)
N1A—C3A	1.4728 (17)	N1B—C3B	1.4751 (17)
C1A—C2A	1.3903 (18)	C1B—C2B	1.3909 (19)
C1A—C6A	1.3919 (18)	C1B—C6B	1.3944 (19)
C1A—H1A	0.93	C1B—H1B	0.93
C2A—C3A	1.3805 (19)	C2B—C3B	1.3808 (19)
C2A—H2A	0.93	C2B—H2B	0.93
C3A—C4A	1.3780 (19)	C3B—C4B	1.380 (2)
C4A—C5A	1.3857 (18)	C4B—C5B	1.3839 (19)
C4A—H4A	0.93	C4B—H4B	0.93
C5A—C6A	1.3944 (18)	C5B—C6B	1.3969 (18)
C5A—H5A	0.93	C5B—H5B	0.93
C6A—C7A	1.5048 (18)	C6B—C7B	1.5039 (18)
C7A—C8A	1.4735 (18)	C7B—C8B	1.4759 (18)
C8A—C9A	1.3422 (18)	C8B—C9B	1.3370 (19)
C8A—H8A	0.93	C8B—H8B	0.93
C9A—C10A	1.4644 (18)	C9B—C10B	1.4594 (18)
C9A—H9A	0.93	C9B—H9B	0.93
C10A—C15A	1.4002 (19)	C10B—C15B	1.3966 (19)
C10A—C11A	1.4024 (18)	C10B—C11B	1.4002 (18)
C11A—C12A	1.3876 (18)	C11B—C12B	1.3903 (18)
C11A—H11A	0.93	C11B—H11B	0.93
C12A—C13A	1.395 (2)	C12B—C13B	1.393 (2)
C12A—H12A	0.93	C12B—H12B	0.93
C13A—C14A	1.3976 (19)	C13B—C14B	1.3992 (19)
C13A—C16A	1.5050 (18)	C13B—C16B	1.5024 (19)
C14A—C15A	1.3853 (18)	C14B—C15B	1.3874 (19)
C14A—H14A	0.93	C14B—H14B	0.93
C15A—H15A	0.93	C15B—H15B	0.93
C16A—H16A	0.96	C16B—H16D	0.96
C16A—H16B	0.96	C16B—H16E	0.96
C16A—H16C	0.96	C16B—H16F	0.96
			
O2A—N1A—O3A	123.99 (12)	O2B—N1B—O3B	124.08 (12)
O2A—N1A—C3A	117.98 (12)	O2B—N1B—C3B	118.32 (12)
O3A—N1A—C3A	118.03 (12)	O3B—N1B—C3B	117.60 (12)
C2A—C1A—C6A	119.95 (12)	C2B—C1B—C6B	120.27 (12)
C2A—C1A—H1A	120.0	C2B—C1B—H1B	119.9
C6A—C1A—H1A	120.0	C6B—C1B—H1B	119.9
C3A—C2A—C1A	118.73 (12)	C3B—C2B—C1B	118.31 (13)
C3A—C2A—H2A	120.6	C3B—C2B—H2B	120.8
C1A—C2A—H2A	120.6	C1B—C2B—H2B	120.8
C4A—C3A—C2A	122.82 (12)	C4B—C3B—C2B	122.99 (12)
C4A—C3A—N1A	119.25 (12)	C4B—C3B—N1B	119.29 (12)
C2A—C3A—N1A	117.92 (12)	C2B—C3B—N1B	117.69 (12)
C3A—C4A—C5A	117.86 (12)	C3B—C4B—C5B	118.12 (12)
C3A—C4A—H4A	121.1	C3B—C4B—H4B	120.9
C5A—C4A—H4A	121.1	C5B—C4B—H4B	120.9
C4A—C5A—C6A	121.03 (13)	C4B—C5B—C6B	120.75 (13)
C4A—C5A—H5A	119.5	C4B—C5B—H5B	119.6
C6A—C5A—H5A	119.5	C6B—C5B—H5B	119.6
C1A—C6A—C5A	119.60 (12)	C1B—C6B—C5B	119.56 (12)
C1A—C6A—C7A	122.29 (12)	C1B—C6B—C7B	122.63 (12)
C5A—C6A—C7A	118.08 (12)	C5B—C6B—C7B	117.79 (12)
O1A—C7A—C8A	122.24 (12)	O1B—C7B—C8B	122.13 (12)
O1A—C7A—C6A	119.41 (12)	O1B—C7B—C6B	119.37 (12)
C8A—C7A—C6A	118.34 (11)	C8B—C7B—C6B	118.50 (12)
C9A—C8A—C7A	120.01 (12)	C9B—C8B—C7B	120.66 (13)
C9A—C8A—H8A	120.0	C9B—C8B—H8B	119.7
C7A—C8A—H8A	120.0	C7B—C8B—H8B	119.7
C8A—C9A—C10A	126.33 (13)	C8B—C9B—C10B	126.94 (13)
C8A—C9A—H9A	116.8	C8B—C9B—H9B	116.5
C10A—C9A—H9A	116.8	C10B—C9B—H9B	116.5
C15A—C10A—C11A	117.72 (12)	C15B—C10B—C11B	118.17 (12)
C15A—C10A—C9A	122.96 (12)	C15B—C10B—C9B	122.17 (12)
C11A—C10A—C9A	119.28 (12)	C11B—C10B—C9B	119.65 (12)
C12A—C11A—C10A	121.57 (13)	C12B—C11B—C10B	120.96 (13)
C12A—C11A—H11A	119.2	C12B—C11B—H11B	119.5
C10A—C11A—H11A	119.2	C10B—C11B—H11B	119.5
C11A—C12A—C13A	120.58 (12)	C11B—C12B—C13B	120.84 (13)
C11A—C12A—H12A	119.7	C11B—C12B—H12B	119.6
C13A—C12A—H12A	119.7	C13B—C12B—H12B	119.6
C12A—C13A—C14A	117.86 (12)	C12B—C13B—C14B	118.14 (12)
C12A—C13A—C16A	121.01 (12)	C12B—C13B—C16B	120.38 (12)
C14A—C13A—C16A	121.13 (13)	C14B—C13B—C16B	121.47 (13)
C15A—C14A—C13A	121.84 (13)	C15B—C14B—C13B	121.19 (13)
C15A—C14A—H14A	119.1	C15B—C14B—H14B	119.4
C13A—C14A—H14A	119.1	C13B—C14B—H14B	119.4
C14A—C15A—C10A	120.40 (12)	C14B—C15B—C10B	120.68 (12)
C14A—C15A—H15A	119.8	C14B—C15B—H15B	119.7
C10A—C15A—H15A	119.8	C10B—C15B—H15B	119.7
C13A—C16A—H16A	109.5	C13B—C16B—H16D	109.5
C13A—C16A—H16B	109.5	C13B—C16B—H16E	109.5
H16A—C16A—H16B	109.5	H16D—C16B—H16E	109.5
C13A—C16A—H16C	109.5	C13B—C16B—H16F	109.5
H16A—C16A—H16C	109.5	H16D—C16B—H16F	109.5
H16B—C16A—H16C	109.5	H16E—C16B—H16F	109.5
			
C6A—C1A—C2A—C3A	−0.6 (2)	C6B—C1B—C2B—C3B	0.8 (2)
C1A—C2A—C3A—C4A	0.4 (2)	C1B—C2B—C3B—C4B	−0.3 (2)
C1A—C2A—C3A—N1A	−179.99 (13)	C1B—C2B—C3B—N1B	177.49 (12)
O2A—N1A—C3A—C4A	−169.19 (14)	O2B—N1B—C3B—C4B	−176.27 (13)
O3A—N1A—C3A—C4A	11.5 (2)	O3B—N1B—C3B—C4B	4.54 (19)
O2A—N1A—C3A—C2A	11.2 (2)	O2B—N1B—C3B—C2B	5.84 (19)
O3A—N1A—C3A—C2A	−168.15 (13)	O3B—N1B—C3B—C2B	−173.35 (13)
C2A—C3A—C4A—C5A	0.0 (2)	C2B—C3B—C4B—C5B	−0.7 (2)
N1A—C3A—C4A—C5A	−179.61 (13)	N1B—C3B—C4B—C5B	−178.43 (12)
C3A—C4A—C5A—C6A	−0.2 (2)	C3B—C4B—C5B—C6B	1.2 (2)
C2A—C1A—C6A—C5A	0.5 (2)	C2B—C1B—C6B—C5B	−0.3 (2)
C2A—C1A—C6A—C7A	−177.56 (13)	C2B—C1B—C6B—C7B	177.65 (13)
C4A—C5A—C6A—C1A	0.0 (2)	C4B—C5B—C6B—C1B	−0.7 (2)
C4A—C5A—C6A—C7A	178.06 (13)	C4B—C5B—C6B—C7B	−178.75 (13)
C1A—C6A—C7A—O1A	159.57 (14)	C1B—C6B—C7B—O1B	−162.74 (14)
C5A—C6A—C7A—O1A	−18.5 (2)	C5B—C6B—C7B—O1B	15.3 (2)
C1A—C6A—C7A—C8A	−21.7 (2)	C1B—C6B—C7B—C8B	17.1 (2)
C5A—C6A—C7A—C8A	160.26 (13)	C5B—C6B—C7B—C8B	−164.91 (12)
O1A—C7A—C8A—C9A	−6.7 (2)	O1B—C7B—C8B—C9B	−3.8 (2)
C6A—C7A—C8A—C9A	174.58 (12)	C6B—C7B—C8B—C9B	176.38 (13)
C7A—C8A—C9A—C10A	174.30 (12)	C7B—C8B—C9B—C10B	179.38 (13)
C8A—C9A—C10A—C15A	−15.3 (2)	C8B—C9B—C10B—C15B	−13.8 (2)
C8A—C9A—C10A—C11A	167.03 (13)	C8B—C9B—C10B—C11B	167.69 (14)
C15A—C10A—C11A—C12A	1.2 (2)	C15B—C10B—C11B—C12B	0.7 (2)
C9A—C10A—C11A—C12A	179.00 (12)	C9B—C10B—C11B—C12B	179.31 (13)
C10A—C11A—C12A—C13A	−1.3 (2)	C10B—C11B—C12B—C13B	−0.7 (2)
C11A—C12A—C13A—C14A	0.1 (2)	C11B—C12B—C13B—C14B	−0.1 (2)
C11A—C12A—C13A—C16A	−179.37 (13)	C11B—C12B—C13B—C16B	179.37 (14)
C12A—C13A—C14A—C15A	1.2 (2)	C12B—C13B—C14B—C15B	0.8 (2)
C16A—C13A—C14A—C15A	−179.32 (13)	C16B—C13B—C14B—C15B	−178.62 (14)
C13A—C14A—C15A—C10A	−1.3 (2)	C13B—C14B—C15B—C10B	−0.8 (2)
C11A—C10A—C15A—C14A	0.09 (19)	C11B—C10B—C15B—C14B	0.0 (2)
C9A—C10A—C15A—C14A	−177.62 (12)	C9B—C10B—C15B—C14B	−178.54 (13)

### Hydrogen-bond geometry (Å, °)

**Table:**

*D*—H···*A*	*D*—H	H···*A*	*D*···*A*	*D*—H···*A*
C1A—H1A···O1B^i^	0.93	2.58	3.2597 (17)	131
C9A—H9A···O1A	0.93	2.48	2.8045 (17)	101
C9B—H9B···O1B	0.93	2.48	2.8112 (17)	101
C1B—H1B···Cg1	0.93	2.90	3.4853 (15)	123
C4B—H4B···Cg1^ii^	0.93	2.86	3.4837 (15)	126
C16A—H16C···Cg2^iii^	0.96	2.91	3.7837 (15)	151

Symmetry codes: (i) *x*+1, *y*−1, *z*;  (ii) *x*−1, *y*+1, *z*; (iii) *x*+1, *y*, *z*.

## References

[bb1] Agrinskaya, N. V., Lukoshkin, V. A., Kudryavtsev, V. V., Nosova, G. I., Solovskaya, N. A. & Yakimanski, A. V. (1999). *Phys. Solid State*, **41**, 1914–1917.

[bb2] Allen, F. H., Kennard, O., Watson, D. G., Brammer, L., Orpen, A. G. & Taylor, R. (1987). *J. Chem. Soc. Perkin Trans. 2*, pp. S1–S19.

[bb3] Bernstein, J., Davis, R. E., Shimoni, L. & Chang, N.-L. (1995). *Angew. Chem. Int. Ed. Engl.* **34**, 1555–1573.

[bb4] Bruker (2005). *APEX2*, *SAINT* and *SADABS* Bruker AXS Inc., Madison, Wisconsin, USA.

[bb5] Fun, H.-K., Patil, P. S., Dharmaprakash, S. M. & Chantrapromma, S. (2007). *Acta Cryst.* E**63**, o561–o562.

[bb6] Gu, B., Ji, W., Patil, P. S., Dharmaprakash, S. M. & Wang, H. T. (2008). *Appl. Phys. Lett.* **92**, 091118.

[bb7] Patil, P. S., Chantrapromma, S., Fun, H.-K. & Dharmaprakash, S. M. (2007*a*). *Acta Cryst.* E**63**, o1738–o1740.

[bb9] Patil, P. S., Dharmaprakash, S. M., Fun, H.-K. & Karthikeyan, M. S. (2006). *J. Cryst. Growth*, **297**, 111–116.

[bb10] Patil, P. S., Dharmaprakash, S. M., Ramakrishna, K., Fun, H.-K., Sai Santosh Kumar, R. & Rao, D. N. (2007). *J. Cryst. Growth*, **303**, 520–524.

[bb8] Patil, P. S., Fun, H.-K., Chantrapromma, S. & Dharmaprakash, S. M. (2007*b*). *Acta Cryst.* E**63**, o2497–o2498.

[bb11] Sheldrick, G. M. (2008). *Acta Cryst.* A**64**, 112–122. 10.1107/S0108767307043930 18156677

[bb12] Spek, A. L. (2003). *J. Appl. Cryst.* **36**, 7–13.

